# Stress-induced loss of social resilience in honeybee colonies and its implications on fitness

**DOI:** 10.1098/rspb.2023.2460

**Published:** 2024-01-10

**Authors:** Zeynep N. Ulgezen, Frank Van Langevelde, Coby van Dooremalen

**Affiliations:** ^1^ Wageningen Plant Research, Wageningen University & Research, Droevendaalsesteeg 1, 6708 PB Wageningen, The Netherlands; ^2^ Wildlife Ecology and Conservation Group, Department of Environmental Sciences, Wageningen University & Research, Droevendaalsesteeg 3a, 6708 PB Wageningen, The Netherlands

**Keywords:** timing of brood rearing, nest emergence, colony fitness, pollen restriction, *Varroa destructor*, *Apis mellifera*

## Abstract

Stressors may lead to a shift in the timing of life-history events of species, causing a mismatch with optimal environmental conditions, potentially reducing fitness. In honeybees, the timing of brood rearing and nest emergence in late winter/early spring is critical as colonies need to grow fast after winter to prepare for reproduction. However, the effects of stress on these life-history events in late winter/early spring and the possible consequences are not well understood. Therefore, we tested whether (i) honeybee colonies shift timing of brood rearing and nest emergence as response to stressors, and (ii) if there is a consequent loss of social resilience, reflected in colony fitness (survival, growth and reproduction). We monitored stressed (high load of the parasitic mite *Varroa destructor* or nutrition restricted) colonies and presumably non-stressed colonies from the beginning of 2020 till spring of 2021. We found that honeybee colonies do not shift the timing of brood rearing and nest emergence in spring as a coping mechanism to stressors. However, we show that there is loss of social resilience in stressed colonies, leading to reduced growth and reproduction. Our study contributes to better understanding the effects of stressors on social resilience in eusocial organisms.

## 1. Introduction

For many species, life-history events, such as the timing of emergence of insects or the migration and breeding of vertebrates, are timed to coincide with the availability of resources or optimal environmental conditions [1]. Shifts in the timing of life-history events compared to for instance the timing of the peak in resources may consequently lead to mismatches. This can reduce fitness and cause population declines [2,3]. The timing of life-history events, and possible mismatches caused by changes in climate and land use, has been of concern in pollinators [4,5], especially due to their role in agriculture and biodiversity, and signs of worldwide decline [6]. Honeybees are one of the major pollinators that have had significant losses caused by multiple stressors [6,7], but possible consequences of mismatches of the timing of life-history events due to these stressors are poorly understood. For honeybees in temperate regions, the transition period from winter to spring is of particular importance as the colony needs to grow rapidly to produce the workforce necessary for reproduction (i.e. swarming). In this paper, we study the potential effects of stressors on the timing of life-history events in this critical time period.

In order to conserve resources, honeybee colonies in temperate regions cease foraging and brood rearing activities prior to or in early winter [8]. Winter bees live throughout winter and collectively participate in the thermoregulation of the colony [9]. Thermoregulation and the broodless state of the colony allow bees to survive through harsh weather conditions [8,10]. Brood rearing and foraging is resumed in late winter by the remaining winter bees in anticipation of resource acquisition and reproduction in spring [11]. These winter bees are replaced by the newly emerging bees, which further contribute to colony development [12]. While previous research with honeybees carried out in climate chambers suggests that these events may be initiated by an interplay between cues of seasonal change, specifically photoperiod and temperature [13] (but see Villagomez *et al*. [14], who did not find an effect of photoperiod and temperature on timing of brood rearing), the main drivers for timing of brood rearing and nest emergence during late winter/early spring are not well understood. It is possible that besides photoperiod and temperature, colony conditions may play a role it in as well, as with honeybee colonies' capability of regulating their activity based on resource availability [15].

Moreover, honeybee colonies can show adaptive responses to harsh conditions and stressors and exhibit behavioural plasticity, where the behaviour of individual bees can be altered to meet colony demands accordingly [16]. For instance, colonies demonstrate division of labour in spring and summer known as temporal polyethism (i.e. switching tasks as they age). The ontogeny of behaviours can be accelerated, delayed or reversed depending on demography [17] and pollen availability [18]. It has also been observed that stressors, such as heat exposure, nutritional restriction, and parasites and pathogens, can alter the ontogeny of behaviours, by pushing bees to switch between tasks faster and start precocious foraging [19–22]. Notably, similar behavioural flexibility has also been observed with regard to life-history events in autumn, where nutritional stress, particularly reducing pollen supplies, can advance the transition of colonies into a broodless state due to the diminished capacity of colonies to produce brood [15]. However, the effects of stressors on timing of brood rearing and nest emergence during late winter/early spring remains unknown. Stressors can lead to shorter lifespan in winter bees [23], smaller colony size [24] and reduced amount of pollen [25]. A lack of resources in the colony, in terms of nutrition and worker bees, may cause a shift in timing of brood rearing and nest emergence in order to preserve or replenish colony resources.

Honeybee colonies are described as superorganisms where interactions among nest-mates and combined individual behaviours maintain the colony at a homeostatic state [26]. The adaptive capacity of superorganisms, that allows them to adjust to stress and provides an ecological buffer against detrimental effects of perturbations is described as social resilience [27,28]. For instance, social immunity contributes to social resilience in colonies, where colonies display behaviours such as corpse removal [29] and grooming [30] to cope with parasites. Considering the flexibility and adaptive responses of colonies, shifting the timing of brood rearing and nest emergence may be used as a coping mechanism to maintain social resilience in the face of stressors [16].

However, shifting the timing of brood rearing and nest emergence can be maladaptive, and possibly lead to a loss in social resilience as resource shortage can lead to a trade-off between colony growth and social resilience. Chronic stressors, such as infestation with the parasitic mite *Varroa destructor*, lead to smaller colony size during winter [24], and such colonies may start brood rearing earlier to compensate and prepare for the growing season. Onset of brood rearing is a major event that requires a sudden surge in energetic demand, and so problems may arise as the colony has to keep the brood at higher temperatures than without brood [31]. In smaller colonies, the energy required for heat production is higher per bee [32]. Energy allocation to brood rearing and thermoregulation may increase the workload and lead to shorter lifespan of individual bees, which further reduces the size of the colony [33]. Previous research shows that inadequate resources in spring can lead to smaller colony sizes [34], and pollen restriction can reduce the amount of brood [35]. These exacerbating circumstances may reduce social resilience and propel a colony into collapse. Postponing the timing of brood rearing and nest emergence may also cause issues as it can hamper the exploitation of spring bloom and impede well-timed reproduction that is synchronous with resource availability. There is evidence which indicates that late onset of brood rearing hinders colony growth and swarming, and that late swarms starve more often during winter compared to early swarms [8].

In this study, we address two questions: (1) Do honeybee colonies adjust the timing of brood rearing and nest emergence in spring as a coping mechanism to stress, and (2) is there a loss of social resilience as a consequence of this shift, specifically on colony survival, growth and reproduction in spring? To answer the first question, we compared the timing of brood rearing and nest emergence between presumably non-stressed and chronically stressed (high infestation with the parasitic mite *Varroa destructor* or nutrition restricted) colonies. We expected that *V. destructor* infested colonies may advance the timing in an attempt to replenish the loss of winter bees. Nutrition restricted colonies may have to delay timing to first gather sufficient amount of resources before starting activities. To answer the second question, we experimentally induced a perturbation after the start of brood rearing to test whether stressed colonies had a loss of social resilience. For this, we subjected the colonies to a cold shock. Theory predicts that an organism loses resilience when there is a slower return rate back to homeostasis after a perturbation, indicated by larger and slower fluctuations in some characteristics of the organism. These larger and slower fluctuations can be measured by the increase in variation [36]. Hence, variance is considered to be a generic indicator of loss of resilience, and has been used as a measure of resilience in diverse complex systems, described as ‘critical slowing down' [37]. Considering its importance in colony homeostasis [38], we used in-hive temperature as a measure for honeybee social resilience as colonies should maintain high nest temperatures (about 35°C) during winter and early spring. Nest temperature is regulated by individual bees that produce heat, and collectively the colony maintains nest temperature at a certain level. Changes in the ambient temperature require a response of the bees by either increasing or decreasing heat production [31]. Honeybees from stressed colonies have lower ability to produce heat and hence are predicted to respond slower to changes in ambient temperature [39]. Our hypothesis was that stressed colonies show higher variance in in-hive temperature due to difficulties in maintaining nest temperature constant, especially after the perturbation compared to non-stressed colonies. Finally, we followed the colonies in spring to investigate the effects of the potential loss of social resilience on survival, growth and reproduction.

## 2. Methods

### (a) Experiment set-up

The experiment took place between January 2020 and May 2021 at an apiary of Wageningen University and Research (51°57′17.7″ N 5°38′12.0″), in the Netherlands. The honeybee colonies (Dutch hybrid of *Apis mellifera* spp.) were supplied by a professional beekeeping company (Inbuzz), and kept in one or two wooden ten-frame hives (inside measures simplex). A queen excluder was placed between boxes when there were two boxes, and the queen was limited to the top box. From November 2020 onwards till the end of the experiment, all hives only had one box. Hives were placed at least 1 m apart and had different colour entrances to minimize drift between colonies. At the start of the experiment, colonies had a standard number of bees and a young (0–1 years) healthy egg-laying queen. All colonies were prevented from swarming during the experiment to keep them intact and prevent disruption of measurements. Sugar dough was fed *ad libitum* throughout the experiment.

Before the start of the experiment, colonies were randomly assigned to either a nutritional stress (pollen restricted)*, V. destructor* (varroa) or control group. Each group consisted of five colonies (*n* = 15). To keep the *V. destructor* infestation low in control and pollen colonies, we treated them with oxalic acid in winter of 2019 and 2020, when there was no brood, by trickling (37 g oxalic acid dihydrate in 1 l sugar water, 1 : 1 weight ratio for sucrose:water), and in summer of 2020 by spraying (30 g oxalic acid dihydrate in 1 l water). Colonies assigned to the varroa group were never treated against mite infestation, from the winter prior to the start until the end of the experiment. Detailed information on treatment validation can be found in the electronic supplementary information.

### (b) Sensor measurements

All colonies were equipped with the BEEPbase Sensor System (https://beep.nl/index.php/measurement-system-2). The BEEPbases included sensors for measuring in-hive temperature (1-Wire, DS18b20) and weighing the hive (Bosche H40A, 0–150 kg + /−10 grams). The weight sensor was placed underneath the hives. For each hive, five in-hive temperature sensors were placed between frames of bees, 9cm deep from the top of the frame; four of them diagonally forming a diamond shape, and one in the centre. Location of temperature sensors was standardized across hives. Measurements were logged in 10 min intervals. We also placed bee counters (bBars, bRemote) at the flight entrance of each hive. The counter recorded the flight activity (i.e. the total number of incoming and outgoing bees) every 10 min for 30 secs. Data were transmitted from a long-range (LoRa) gateway. The BEEP app (https://beep.nl/index.php/beep-app) was used to record automatically acquired data from the sensors. Automated measurements started in June of 2020.

### (c) Timing of brood rearing and nest emergence

For the analysis of the timing of brood rearing, we used the mean daily in-hive temperature from January 2021 onwards. The mean per day was calculated by using the maximum in-hive temperature recorded every 10 min (the one sensor out of five with the highest temperature value), irrespective of the location of the sensors. The specific sensor that recorded the maximum temperature was highly consistent over time, suggesting that the bee cluster and brood did not move often, and that the placement of sensors captured the bee cluster and brood temperature (see electronic supplementary material). Given that brood rearing requires constant temperatures of about 35°C (compared to in-hive temperature of around 21°C in the absence of brood [31]), the start of brood rearing was determined via graphical representation of the mean in-hive temperature over time. The first day in the leap in in-hive temperature from one stage to the next was presumed as the initiation of brood rearing and used in the analysis. The presence of brood was confirmed by visual inspections of colonies.

The foraging activity data from bee counters was used as a proxy to investigate the timing of nest emergence. We presumed that emergence starts when more forager bees will fly out and continue to fly out to collect resources needed to feed the rapidly increasing number of larvae and young bees in the brood nest (reviewed in [40]). The total activity (incoming and outgoing bees) per day was used for the analysis. To compare the differences between treatments, we used a linear mixed model (LMM) with treatment, day and their interaction as fixed factor and colony as subject to account for repeated measurements. Due to a technical failure of the bee counters, there was data missing from the beginning of 2021 and for several days later in the year. The days that the hives were opened were also excluded from the analysis. As we were interested in the onset of brood rearing and nest emergence, we only used data from 2021.

### (d) Social resilience

To test whether stressed colonies had a loss of social resilience, we subjected the colonies to the cold shock experiment in the first week of March 2021. The experiment was performed in three batches over three consecutive nights, where per batch five different colonies (randomly assigned from different treatment groups) were placed in a −20°C freezer between 17.00 and 08.30. The cold shock lasted for 15.5 h for all colonies (mean ± s.d. = 15 h 30 min ± 2 min). At the time of the cold shock, all colonies had brood present.

Social resilience was measured by calculating the variance of the in-hive temperature per colony, using the mean daily temperature. As only one temperature sensor per hive recorded the maximum temperature most frequently, we presume that the variance is not an artefact of the movement of the bee cluster and brood (see electronic supplementary material). To compare treatments, we used Levene's test for equality of variances for three different time periods: (i) pre-brood (January 2021 till the start of brood rearing; (ii) brood (start of brood rearing till cold shock experiment); and (iii) post-cold shock (after the cold shock experiment till the end of measurements). We expect the variance of the in-hive temperature to increase after the cold shock, especially compared to the period that the colonies had brood. Pairwise comparison was done between each pair of treatments within every time period using a Bonferroni correction.

### (e) Colony size and brood size during spring

We estimated colony size throughout winter 2020–2021 and spring of 2021. Here, we present the colony size and number of brood cells during spring 2021 (March 2021–May 2021) for analysing colony growth post winter. Information on the colony size and bee survival during winter is given in the electronic supplementary material. Colony size was measured using photo analysis, weather permitting, once a month in 2020, and every two weeks from March 2021 onwards, following the same methods described in van Dooremalen *et al*. [27]. Photos of the hive were taken from the top. The number of bees in the colony was calculated by using the fraction of bees (area occupied by bees divided by the area available). For the number of brood cells, the area of brood in all stages (pupae, larvae and eggs) was estimated by placing a grid (5 cm × 5 cm squares) over each side of every frame and counting the number of squares. The values were summed and a factor of 4 cells per cm^2^ was used to calculate the total number of brood cells [41]. The total number of worker brood cells per colony was used for analysis. Measurements were done twice in spring of 2021 (March and April).

Treatments were compared by using LMMs with estimates of colony size and total number of brood cells as dependent variables. Treatment, month and their interaction were included as fixed factor, and colony as subject for repeated measurements. Tukey's HSD was used for comparisons between means of different treatments.

### (f) Colony reproduction and survival

As the colonies were restricted from swarming, the number of drone brood cells was used as a proxy to compare colony reproduction between treatments. Swarming has been positively associated both with the presence and amount of drone brood present [42]. Start of drone brood presence was checked weekly from March till May 2021. The number of drone brood cells was measured using the same method described for estimating worker brood. Since no colonies had drone brood present in March, only data from April was used for the analysis.

To test for differences in the number of drone brood cells between treatments, we used a Kruskal–Wallis non-parametric test. Dunn's test was used for pairwise comparisons between treatments. For the start of drone brood rearing, we did not use a statistical analysis to test for differences between treatments due to low and unequal sample sizes. Prior to the start of drone brood rearing four colonies collapsed (three varroa and one pollen), and one pollen colony failed to start drone rearing till the time the experiments were concluded (May 2021).

## 3. Results

### (a) Timing of brood rearing and nest emergence

**Figure 1.**
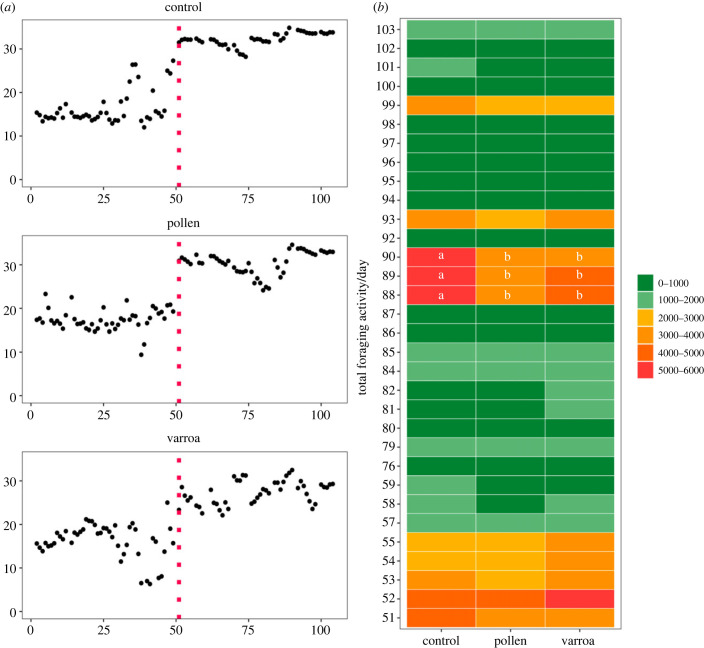
Timing of brood rearing and nest emergence in colonies. (*a*) Examples of in-hive temperature over time (day of year). From top to bottom: control (presumably healthy), pollen (pollen restricted) and varroa (high *V. destructor* infested) colonies. The vertical dashed line represents the start of brood rearing. (*b*) Mean total foraging activity (used as proxy for nest emergence) of all colonies per day per treatment. Letters indicate significant differences between treatments within days. No letters mean no significant differences were found. Day 1 is 1 January 2021.

No differences were found regarding the timing of brood rearing between the different treatments. Almost all colonies started brood rearing on the same day of the year, day 51 (mean = 51.2, s.d. = 0.1 days). Three colonies started brood rearing on day 52. Figure 1*a* shows examples of in-hive temperature over-time, starting at 1st January, from the three different treatments. See the electronic supplementary material for mean in-hive temperature per day of all colonies.

Similar to the start of brood rearing, no shift in timing of nest emergence was found in the stressed colonies compared to the control colonies. While there was a difference between treatments in foraging activity (LMM: treatment * day *F*_62,465_ = 1.58, *p* = 0.005), this was only reflected between some days later in the year (days 88–90), where control colonies showed higher activity compared to stressed colonies (figure 1*b*).

### (b) Social resilience

**Figure 2.**
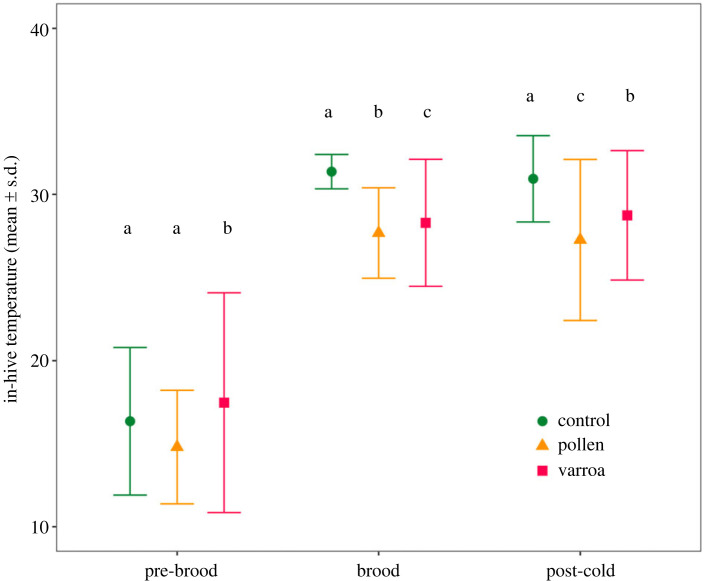
Mean temperature and standard deviation of the in-hive temperature before the start of brood rearing (pre-brood, colonies in winter modus), after start of brood rearing (brood) and post-cold shock experiment (post-cold) per treatment. For the statistical analysis, we used the variance of the in-hive temperature as proxy for social resilience of the colonies. Letters indicate significant differences between the variance in in-hive temperature of treatments within each period based on the pairwise comparisons with Bonferroni correction.

Chronic stress and the cold shock had an effect on thermoregulation ability of colonies. The variances of in-hive temperature were unequal between treatments in all time periods (Levene's test: pre-brood *F*_2,662_ = 39.80, *p* < 0.001; brood *F*_2,130_ = 26.04, *p* < 0.001; post-cold shock *F*_2,534_ = 26.77, *p* < 0.001). Stressed colonies generally had a higher variance compared to control colonies in all time periods (figure 2). Before the start of brood rearing, varroa colonies had a significantly higher variance compared to other treatments (control-varroa *p* < 0.001; pollen-varroa *p* < 0.001), while control and pollen colonies did not differ (*p* = 0.054). After the start of brood rearing, control colonies had the lowest variance in comparison, and differed from both pollen (*p* < 0.001) and varroa (*p* < 0.001) colonies. Between stressed colonies (pollen and varroa), varroa once again had the highest variance (*p* < 0.001). After the cold shock experiment, control colonies had the lowest variance (control-varroa *p* < 0.001; control-pollen *p* < 0.001). Differing from previous periods, pollen colonies had the highest variance (varroa-pollen *p* < 0.001). While we did not test for differences between periods, variance seemed to be generally higher in the pre-brood and post-cold shock period.

### (c) Colony size, brood size and reproduction in spring

**Figure 3.**
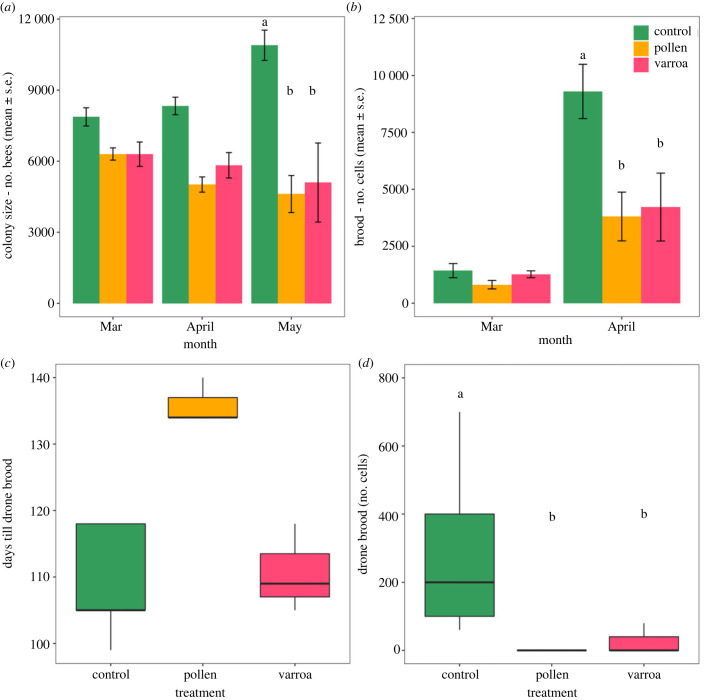
Colony size, brood size and reproduction during spring of 2021. (*a*) Estimated mean number of bees over spring months for each treatment. (*b*) Mean number of brood cells over spring months for each treatment. (*c*) Onset of drone brood rearing (as a proxy for reproduction) in spring for each treatment. (*b*) Number of drone brood cells per treatment in April 2021. Letters indicate significant differences between treatments within months. No letters mean no significant differences were found.

Chronic stress had an effect on the growth of colonies in spring, especially as the months progressed from March to May in 2021. This was seen in both colony size (LMM: treatment × month *F*_4,150_ = 7.2, *p* < 0.001) and number of brood cells (LMM: treatment × month *F*_2,30_ = 6.07, *p* = 0.006). Colony size was largest in control colonies throughout and increased over time, and showed a significant difference compared to stressed colonies in May (pollen–control *p* < 0.001; control–varroa *p* = 0.002) (figure 3*a*). There were no large differences between colony size of varroa and pollen colonies. The number of brood cells was low in all colonies in March. However, control colonies had a significantly higher number of brood cells in April, compared to chronically stressed colonies (control–varroa *p* < 0.001; control–pollen *p* < 0.001) (figure 3*b*). There were no differences between the two different treatments of stressed colonies (*p* = 0.9). Results on colony size and bee survival during winter are given in the electronic supplementary material.

Stressed colonies were more likely to have reproductive issues, delay or failure compared to control colonies. Notably, several stressed colonies collapsed (three varroa and one pollen colonies) or failed to start reproduction (one pollen colony) within the period of the experiment. Our data suggest that stressed colonies, especially those that are pollen restricted, start drone brood rearing at a later date (figure 3*c*). For the colonies that did start brood rearing, control colonies had a higher number of drone brood cells (Kruskal–Wallis: treatment *H* = 10.87, d.f. = 2, *p* = 0.004) compared to both varroa (*p* = 0.028) and pollen colonies (*p* = 0.004) (figure 3*d*).

## 4. Discussion

In this study, we investigated the effects of stress on the timing of life-history events and the social resilience of honeybee colonies in spring. Honeybee colonies can show high behavioural flexibility in response to stressors, such as accelerated worker maturation when exposed to heat [22] or to parasite and pathogens [19–21], and earlier transitioning into winter state due to diminished food resources [15]. Therefore, we hypothesized that colonies may shift timing of brood rearing and nest emergence in late winter/early spring to cope with stressors. Contrary to our expectations all colonies started brood rearing almost on the exact same date, and we did not observe any notable differences between timing of nest emergence. Yet, importantly, we show colonies lose social resilience in response to stressors, and suffer with regard to spring growth and reproduction.

### (a) Social resilience relates to colony fitness

Here, we demonstrated that there is a measurable loss of social resilience in honeybee colonies, as seen by the larger variance of in-hive temperature in chronically stressed colonies compared to control colonies. Overall there was a large variance prior to start of brood rearing (pre-brood period), as temperature in colonies is much more variable in the absence of brood [43]. The difference in variance was especially apparent after brood rearing initiated (brood period) and there was a notable increase in variance after the cold shock experiment. This suggests a loss of social resilience in stressed colonies.

Thermal homeostasis of honeybee colonies has been well studied [44], and has been associated with colony health. For example, Meikle *et al*. [45] showed within-day in-hive temperature variability was higher among colonies exposed to commercial agriculture compared to hives kept near natural forage, indicating reduced temperature control. Previous studies mention colony resilience in context of stressors [16,27], but there has not been any research measuring and comparing the resilience between chronically stressed and non-stressed colonies. In honeybee research, classic measurements on effects of stress and colony performance, for instance colony size, brood size and parasite load [41,46] have been labour-intensive and, more importantly, disruptive to honeybee colonies.

In recent years, there has been a shift towards exploring the possible uses of automated sensors to collect non-intrusive, high resolution and continuous data on colony characteristics [47] and predict colony health [48,49]. These technological developments that are conducive to time-series data can also be used to measure social resilience of honeybee colonies, which may be a valuable indicator of colony fitness [36]. Variance of in-hive temperature is a promising measure of social resilience due to temperature's role in nest homeostasis [33]. Furthermore, an increase in variance is a well-known generic indicator of loss of resilience [36,50,51] and has had universal applications in diverse systems, including measuring health and fertility of cows [52], rainforest dieback and vegetation [53], and mental health of humans [54]. This supports our use of variance in in-hive temperature as indicator for honeybee colony social resilience.

### (b) Causes of loss of social resilience

The increase in variance of in-hive temperature after the start of brood rearing in stressed colonies may be caused by several reasons. Colony size is a crucial factor in superorganism resilience [55]. It has been suggested that as colony size decreases, the higher energy demand per bee may shorten lifespan of individuals and trigger a positive feedback in the colony, resulting in a further decrease in colony size, and eventually lead to collapse [33]. Not much is known about the effect of nutritional stress on colony size in winter, but several studies suggest there is a positive link between amount of pollen stored and colony size [56,57]. Here, we show that pollen restriction throughout summer leads to a reduced colony size late winter/early spring (see also electronic supplementary material), which may have caused the loss of social resilience. While we could not measure the lifespan of bees in pollen restricted colonies due to time limitations, nutritional stress has been linked with shorter lifespan [58,59] which may have led to smaller colony size [24]. *Varroa destructor* infestation in our study led to shorter lifespan of bees (see electronic supplementary material), which supports earlier findings [24,60]. The reduced lifespan of *V. destructor* infested individuals may have a more pronounced, but delayed, effect on colony size [24] in terms of spring development, reproduction and survival.

### (c) Consequences of loss of social resilience

Both *V. destructor* infested and pollen restricted colonies, alongside smaller colony sizes, also had lower number of brood cells in early spring. Previous studies found similar results where reducing pollen resources in spring has been associated with smaller brood size and colony size later in the season [61]. The reduced spring growth in stressed colonies may have been influenced by the thermal instability during the presence of brood. Ambient temperatures can be low in early spring, which already makes maintaining thermal homeostasis a challenge for colonies. Stress exposure can exacerbate circumstances and lead to subpar thermoregulation in the colony, causing adverse effects on colony fitness. Brood reared at lower temperature hatch later [62] and as adults may have issues with behavioural performance [63,64] and lower survival [62,65]. Low temperature has also been shown to negatively affect queen sperm viability [66], which has been linked to colony performance [67]. The high temperature variability in our stressed colonies suggest suboptimal temperatures. Therefore, coupled with the already smaller size of stressed colonies, the loss of social resilience during late winter/early spring may have impeded growth to compensate for the small colony size.

Our results also suggest that the diminished growth in colonies lead to reproductive delay or failure of the colony, or even colony loss. Previous results support this finding, where colony size in spring has been linked with a higher probability of reproduction [68] and spring survival [69]. Several stressed colonies in our experiment collapsed prior to start of reproduction. The stressed colonies that did show onset of drone brood rearing, started reproduction at a later date, especially in pollen restricted colonies. These colonies also had a lower number of drone brood cells. In temperate regions, spring is a time of fast growth for honeybee colonies to prepare for colony reproduction. The trade-off between growth and maintaining social resilience can leave colonies exposed and more vulnerable to effects of perturbations.

### (d) Possible factors determining timing of brood rearing and nest emergence

Almost all colonies started brood rearing on the same day, suggesting the presence of strong climatological triggers for timing of these life-history events in late winter/early spring for honeybee colonies. The similarities we found in the pattern between climatological factors, especially irradiance and ambient temperature, and in-hive temperature support this hypothesis (see electronic supplementary material). However, our colonies were all located in one apiary and we measured the timing of brood rearing and nest emergence only within one year. Therefore, we are unable to test the influence of external drivers on the start of these life-history events. The triggers for timing of brood rearing and nest emergence remain ambiguous and should be studied further.

### (e) Concluding remarks

Our results indicate that honeybee colonies do not shift the timing of the life-history events, brood rearing and nest emergence, in spring as a coping mechanism to stressors. However, we found that there is a loss of social resilience in chronically stressed colonies, as seen by loss of thermal homeostasis leading to stunted growth, diminished reproduction and even colony loss. The results of our study contribute to better understanding of the effects of stressors on social resilience in eusocial organisms. With the accessibility of technology and rise in developments for use in ecology [70], non-intrusive measures of fitness such as variance in in-hive temperature can be easily implemented. As we only focused on the effects of stressors on the timing of life-history events and social resilience in spring, we suggest that future research, for a more comprehensive understanding on social resilience, should focus on the applicability of generic indicators of resilience as a measure of colony fitness throughout the honeybee colony life cycle.

## Data Availability

Data available from Dryad Digital Repository [71], and in the electronic supplementary material [72].

## References

[1] Zhemchuzhnikov MK, Versluijs TSL, Lameris TK, Reneerkens J, Both C, Van Gils JA. 2021 Exploring the drivers of variation in trophic mismatches: a systematic review of long-term avian studies. Ecol. Evol. **11**, 3710-3725. (10.1002/ece3.7346) 33976770 PMC8093693

[2] Both C, Bouwhuis S, Lessells C, Visser ME. 2006 Climate change and population declines in a long-distance migratory bird. Nature **441**, 81-83. (10.1038/nature04539) 16672969

[3] Lameris TK, van der Jeugd HP, Eichhorn G, Dokter AM, Bouten W, Boom MP, Litvin KE, Ens BJ, Nolet BA. 2018 Arctic geese tune migration to a warming climate but still suffer from a phenological mismatch. Curr. Biol. **28**, 2467-2473. (10.1016/j.cub.2018.05.077) 30033332

[4] Memmott J, Craze PG, Waser NM, Price MV. 2007 Global warming and the disruption of plant–pollinator interactions. Ecol. Lett. **10**, 710-717. (10.1111/j.1461-0248.2007.01061.x) 17594426

[5] Scheffers BR et al. 2016 The broad footprint of climate change from genes to biomes to people. Science **354**, aaf7671. (10.1126/science.aaf7671) 27846577

[6] Potts SG, Biesmeijer JC, Kremen C, Neumann P, Schweiger O, Kunin WE. 2010 Global pollinator declines: trends, impacts and drivers. Trends Ecol. Evol. **25**, 345-353. (10.1016/j.tree.2010.01.007) 20188434

[7] Sánchez-Bayo F, Wyckhuys KA. 2019 Worldwide decline of the entomofauna: a review of its drivers. Biol. Conserv. **232**, 8-27. (10.1016/j.biocon.2019.01.020)

[8] Seeley TD, Visscher PK. 1985 Survival of honeybees in cold climates: the critical timing of colony growth and reproduction. Ecol. Entomol. **10**, 81-88. (10.1111/j.1365-2311.1985.tb00537.x)

[9] Southwick EE. 1983 The honey bee cluster as a homeothermic superorganism. Comparat. Biochem. Physiol. A: Physiol. **75**, 641-645. (10.1016/0300-9629(83)90434-6)

[10] Southwick EE. 1985 Allometric relations, metabolism and heart conductance in clusters of honey bees at cool temperatures. J. Comparat. Physiol. B **156**, 143-149. (10.1007/BF00692937)

[11] Seeley TD. 1985 Honeybee ecology: a study of adaptation in social life. Princeton, NJ: Princeton University Press.

[12] Johnson BR. 2010 Division of labor in honeybees: form, function, and proximate mechanisms. Behav. Ecol. Sociobiol. **64**, 305-316. (10.1007/s00265-009-0874-7) 20119486 PMC2810364

[13] Nürnberger F, Härtel S, Steffan-Dewenter I. 2018 The influence of temperature and photoperiod on the timing of brood onset in hibernating honey bee colonies. PeerJ **6**, e4801. (10.7717/peerj.4801) 29844964 PMC5971834

[14] Villagomez GN, Nürnberger F, Requier F, Schiele S, Steffan-Dewenter I. 2021 Effects of temperature and photoperiod on the seasonal timing of Western honey bee colonies and an early spring flowering plant. Ecol. Evol. **11**, 7834-7849. (10.1002/ece3.7616) 34188855 PMC8216905

[15] Mattila HR, Otis GW. 2007 Dwindling pollen resources trigger the transition to broodless populations of long-lived honeybees each autumn. Ecol. Entomol. **32**, 496-505. (10.1111/j.1365-2311.2007.00904.x)

[16] Ulgezen ZN, van Dooremalen C, van Langevelde F. 2021 Understanding social resilience in honeybee colonies. Curr. Res. Insect Sci. **1**, 100021. (10.1016/j.cris.2021.100021) 36003609 PMC9387495

[17] Huang Z-Y, Robinson GE. 1996 Regulation of honey bee division of labor by colony age demography. Behav. Ecol. Sociobiol. **39**, 147-158. (10.1007/s002650050276)

[18] Fewell JH, Winston ML. 1992 Colony state and regulation of pollen foraging in the honey bee, *Apis mellifera* L. Behav. Ecol. Sociobiol. **30**, 387-393. (10.1007/BF00176173)

[19] Dussaubat C, Maisonnasse A, Crauser D, Beslay D, Costagliola G, Soubeyrand S, Kretzchmar A, Le Conte Y. 2013 Flight behavior and pheromone changes associated to Nosema ceranae infection of honey bee workers (Apis mellifera) in field conditions. J. Invertebr. Pathol. **113**, 42-51. (10.1016/j.jip.2013.01.002) 23352958

[20] Janmaat A, Winston M. 2000 The influence of pollen storage area and *Varroa jacobsoni* Oudemans parasitism on temporal caste structure in honey bees (*Apis mellifera* L.). Insectes Soc. **47**, 177-182. (10.1007/PL00001698)

[21] Goblirsch M, Huang ZY, Spivak M. 2013 Physiological and behavioral changes in honey bees (*Apis mellifera*) induced by *Nosema ceranae* infection. PLoS ONE **8**, e58165. (10.1371/journal.pone.0058165) 23483987 PMC3590174

[22] Bordier C, Suchail S, Pioz M, Devaud JM, Collet C, Charreton M, Le Conte Y, Alaux C. 2017 Stress response in honeybees is associated with changes in task-related physiology and energetic metabolism. J. Insect. Physiol. **98**, 47-54. (10.1016/j.jinsphys.2016.11.013) 27908721

[23] Amdam GV, Hartfelder K, Norberg K, Hagen A, Omholt SW. 2004 Altered physiology in worker honey bees (Hymenoptera: Apidae) infested with the mite *Varroa destructor* (Acari: Varroidae): a factor in colony loss during overwintering? J. Econ. Entomol. **97**, 741-747. (10.1093/jee/97.3.741) 15279246

[24] Van Dooremalen C, Gerritsen L, Cornelissen B, van der Steen JJ, van Langevelde F, Blacquiere T. 2012 Winter survival of individual honey bees and honey bee colonies depends on level of Varroa destructor infestation. PLoS ONE **7**, e36285. (10.1371/journal.pone.0036285) 22558421 PMC3338694

[25] Wu-Smart J, Spivak M. 2016 Sub-lethal effects of dietary neonicotinoid insecticide exposure on honey bee queen fecundity and colony development. Sci. Rep. **6**, 1-11. (10.1038/srep32108) 27562025 PMC4999797

[26] Schmickl T, Crailsheim K. 2004 Inner nest homeostasis in a changing environment with special emphasis on honey bee brood nursing and pollen supply. Apidologie **35**, 249-263. (10.1051/apido:2004019)

[27] van Dooremalen C, Cornelissen B, Poleij-Hok-Ahin C, Blacquière T. 2018 Single and interactive effects of *Varroa destructor*, Nosema spp., and imidacloprid on honey bee colonies (*Apis mellifera*). Ecosphere **9**, e02378. (10.1002/ecs2.2378)

[28] Sendova-Franks AB, Franks NR. 1994 Social resilience in individual worker ants and its role in division of labour. Proc. R. Soc. Lond. B **256**, 305-309. (10.1098/rspb.1994.0085)

[29] van Langevelde F, Kiggen F, van Dooremalen C, Cornelissen B. 2020 Corpse removal increases when honey bee colonies experience high *Varroa destructor* infestation. Insectes Soc. **67**, 507-513. (10.1007/s00040-020-00789-y)

[30] Kruitwagen A, van Langevelde F, van Dooremalen C, Blacquière T. 2017 Naturally selected honey bee (*Apis mellifera*) colonies resistant to Varroa destructor do not groom more intensively. J. Apicult. Res. **56**, 354-365. (10.1080/00218839.2017.1329797)

[31] Fahrenholz L, Lamprecht I, Schricker B. 1989 Thermal investigations of a honey bee colony: thermoregulation of the hive during summer and winter and heat production of members of different bee castes. J. Comp. Physiol. B **159**, 551-560. (10.1007/BF00694379)

[32] Stabentheiner A, Pressl H, Papst T, Hrassnigg N, Crailsheim K. 2003 Endothermic heat production in honeybee winter clusters. J. Exp. Biol. **206**, 353-358. (10.1242/jeb.00082) 12477904

[33] Bastiaansen R, Doelman A, Van Langevelde F, Rottschafer V. 2020 Modeling honey bee colonies in winter using a Keller–Segel model with a sign-changing chemotactic coefficient. SIAM J. Appl. Math. **80**, 839-863. (10.1137/19M1246067)

[34] Farrar C. 1936 Influence of pollen reserves on the surviving populations of over-wintered colonies. Am. Bee J. **76**, 452-454.

[35] Schmickl T, Crailsheim K. 2001 Cannibalism and early capping: strategy of honeybee colonies in times of experimental pollen shortages. J. Comp. Physiol. **187**, 541-547. (10.1007/s003590100226) 11730301

[36] Scheffer M et al. 2018 Quantifying resilience of humans and other animals. Proc. Natl Acad. Sci. USA **115**, 11 883-11 890. (10.1073/pnas.1810630115) PMC6255191 30373844

[37] Dakos V et al. 2012 Methods for detecting early warnings of critical transitions in time series illustrated using simulated ecological data. PLoS ONE **7**, e41010. (10.1371/journal.pone.0041010) 22815897 PMC3398887

[38] Human H, Nicolson SW, Dietemann V. 2006 Do honeybees, *Apis mellifera* scutellata, regulate humidity in their nest? Naturwissenschaften **93**, 397-401. (10.1007/s00114-006-0117-y) 16670906

[39] van Langevelde F, Ulgezen ZN, Oteman B, van Dooremalen C. 2023 Collapse of social resilience in honeybee colonies during winter. *Manuscript in Preparation*.

[40] Knoll S, Pinna W, Varcasia A, Scala A, Cappai MG 2020 The honey bee (*Apis mellifera* L., 1758) and the seasonal adaptation of productions. Highlights on summer to winter transition and back to summer metabolic activity. A review. Livestock Sci. **235**, 104011.

[41] Delaplane KS, Van Der Steen J, Guzman-Novoa E. 2013 Standard methods for estimating strength parameters *of Apis mellifera* colonies. J. Apicul. Res. **52**, 1-12. (10.3896/IBRA.1.52.1.03)

[42] Allsopp M, Hepburn H. 1997 Swarming, supersedure and the mating system of a natural population of honey bees (*Apis mellifera* capensis). J. Apicul. Res. **36**, 41-48. (10.1080/00218839.1997.11100929)

[43] Stabentheiner A, Kovac H, Brodschneider R. 2010 Honeybee colony thermoregulation–regulatory mechanisms and contribution of individuals in dependence on age, location and thermal stress. PLoS ONE **5**, e8967. (10.1371/journal.pone.0008967) 20126462 PMC2813292

[44] Stabentheiner A, Kovac H, Mandl M, Käfer H. 2021 Coping with the cold and fighting the heat: thermal homeostasis of a superorganism, the honeybee colony. J. Comp. Physiol. A **207**, 337-351. (10.1007/s00359-021-01464-8) PMC8079341 33598719

[45] Meikle WG, Weiss M, Maes PW, Fitz W, Snyder LA, Sheehan T, Mott BM, Anderson KE. 2017 Internal hive temperature as a means of monitoring honey bee colony health in a migratory beekeeping operation before and during winter. Apidologie **48**, 666-680. (10.1007/s13592-017-0512-8)

[46] Dietemann V et al. 2013 Standard methods for varroa research. J. Apicul. Res. **52**, 1-54. (10.3896/IBRA.1.52.1.09)

[47] Meikle W, Holst N. 2015 Application of continuous monitoring of honeybee colonies. Apidologie **46**, 10-22. (10.1007/s13592-014-0298-x)

[48] Braga AR, Gomes DG, Rogers R, Hassler EE, Freitas BM, Cazier JA. 2020 A method for mining combined data from in-hive sensors, weather and apiary inspections to forecast the health status of honey bee colonies. Comput. Electron. Agric. **169**, 105161. (10.1016/j.compag.2019.105161)

[49] Cecchi S, Spinsante S, Terenzi A, Orcioni S. 2020 A smart sensor-based measurement system for advanced bee hive monitoring. Sensors **20**, 2726. (10.3390/s20092726) 32397686 PMC7248914

[50] Carpenter SR, Brock WA. 2006 Rising variance: a leading indicator of ecological transition. Ecol. Lett. **9**, 311-318. (10.1111/j.1461-0248.2005.00877.x) 16958897

[51] Scheffer M et al. 2009 Early-warning signals for critical transitions. Nature **461**, 53-59. (10.1038/nature08227) 19727193

[52] Poppe M, Veerkamp R, Van Pelt M, Mulder H. 2020 Exploration of variance, autocorrelation, and skewness of deviations from lactation curves as resilience indicators for breeding. J. Dairy Sci. **103**, 1667-1684. (10.3168/jds.2019-17290) 31759590

[53] Boulton CA, Lenton TM, Boers N. 2022 Pronounced loss of Amazon rainforest resilience since the early 2000s. Nat. Clim. Change **12**, 271-278. (10.1038/s41558-022-01287-8)

[54] Wichers M, Groot PC. 2016 Critical slowing down as a personalized early warning signal for depression. Psychother. Psychosom. **85**, 114-116. (10.1159/000441458) 26821231

[55] Straub L, Williams GR, Pettis J, Fries I, Neumann P. 2015 Superorganism resilience: eusociality and susceptibility of ecosystem service providing insects to stressors. Curr. Opin. Insect Sci. **12**, 109-112. (10.1016/j.cois.2015.10.010)

[56] Brodschneider R, Crailsheim K. 2010 Nutrition and health in honey bees. Apidologie **41**, 278-294. (10.1051/apido/2010012)

[57] Keller I, Fluri P, Imdorf A. 2005 Pollen nutrition and colony development in honey bees—Part II. Bee World **86**, 27-34. (10.1080/0005772X.2005.11099650)

[58] Mattila H, Otis G. 2006 Influence of pollen diet in spring on development of honey bee (Hymenoptera: Apidae) colonies. J. Econ. Entomol. **99**, 604-613. (10.1093/jee/99.3.604) 16813288

[59] Scofield HN, Mattila HR. 2015 Honey bee workers that are pollen stressed as larvae become poor foragers and waggle dancers as adults. PLoS ONE **10**, e0121731. (10.1371/journal.pone.0121731) 25853902 PMC4390236

[60] Dainat B, Evans JD, Chen YP, Gauthier L, Neumann P. 2012 Dead or alive: deformed wing virus and Varroa destructor reduce the life span of winter honeybees. Appl. Environ. Microbiol. **78**, 981-987. (10.1128/AEM.06537-11) 22179240 PMC3273028

[61] Requier F, Odoux JF, Henry M, Bretagnolle V. 2017 The carry-over effects of pollen shortage decrease the survival of honeybee colonies in farmlands. J. Appl. Ecol. **54**, 1161-1170. (10.1111/1365-2664.12836)

[62] Ramirez L, Negri P, Sturla L, Guida L, Vigliarolo T, Maggi M, Eguaras M, Zocchi E, Lamattina L. 2017 Abscisic acid enhances cold tolerance in honeybee larvae. Proc. R. Soc. B **284**, 20162140. (10.1098/rspb.2016.2140) PMC5394655 28381619

[63] Tautz J, Maier S, Groh C, Rössler W, Brockmann A. 2003 Behavioral performance in adult honey bees is influenced by the temperature experienced during their pupal development. Proc. Natl Acad. Sci. USA **100**, 7343-7347. (10.1073/pnas.1232346100) 12764227 PMC165877

[64] Becher MA, Scharpenberg H, Moritz RF. 2009 Pupal developmental temperature and behavioral specialization of honeybee workers (*Apis mellifera* L). J. Comp. Physiol. A **195**, 673-679. (10.1007/s00359-009-0442-7) 19390855

[65] Wang Q, Xu X, Zhu X, Chen L, Zhou S, Huang ZY, Zhou B. 2016 Low-temperature stress during capped brood stage increases pupal mortality, misorientation and adult mortality in honey bees. PLoS ONE **11**, e0154547. (10.1371/journal.pone.0154547) 27149383 PMC4858150

[66] McAfee A, Chapman A, Pettis JS, Foster LJ, Tarpy DR. 2021 Trade-offs between sperm viability and immune protein expression in honey bee queens (*Apis mellifera*). Commun. Biol. **4**, 48. (10.1038/s42003-020-01586-w) 33420325 PMC7794525

[67] Pettis JS, Rice N, Joselow K, van Engelsdorp D, Chaimanee V. 2016 Colony failure linked to low sperm viability in honey bee (*Apis mellifera*) queens and an exploration of potential causative factors. PLoS ONE **11**, e0147220. (10.1371/journal.pone.0147220) 26863438 PMC4749221

[68] Smith ML, Ostwald MM, Loftus JC, Seeley TD. 2014 A critical number of workers in a honeybee colony triggers investment in reproduction. Naturwissenschaften **101**, 783-790. (10.1007/s00114-014-1215-x) 25142633

[69] Harbo JR. 1986 Effect of population size on brood production, worker survival and honey gain in colonies of honeybees. J. Apicul. Res. **25**, 22-29. (10.1080/00218839.1986.11100687)

[70] Tuia D et al. 2022 Perspectives in machine learning for wildlife conservation. Nat. Commun. **13**, 792. (10.1038/s41467-022-27980-y) 35140206 PMC8828720

[71] Ulgezen ZN, van Langevelde F, van Dooremalen C. 2023 Data from: Stress-induced loss of social resilience in honeybee colonies and its implications on fitness. Dryad Digital Repository. (10.5061/dryad.2280gb60c) PMC10777151 38196354

[72] Ulgezen ZN, van Langevelde F, van Dooremalen C. 2023 Stress-induced loss of social resilience in honeybee colonies and its implications on fitness. Figshare. (10.6084/m9.figshare.c.6992006) PMC10777151 38196354

